# Well-Being and Healthcare Inequality on Bulon-Don Island in Southern Thailand—Results of a Pre-Intervention Field Survey

**DOI:** 10.3390/children11101217

**Published:** 2024-10-06

**Authors:** Chutarat Sathirapanya, Suweena Khwanmad, Pornchai Sathirapanya

**Affiliations:** 1 Department of Family and Preventive Medicine, Faculty of Medicine, Prince of Songkla University, Hat Yai 90110, Songkhla, Thailand; 2 Research Center for Kids and Youth Development, Faculty of Medicine, Prince of Songkla University, Hat Yai 90110, Songkhla, Thailand; 3 Sumnakkham Subdistrict Municipality, Sadoa 90320, Songkhla, Thailand; h.mudlah@gmail.com; 4 Department of Medicine, Faculty of Medicine, Prince of Songkla University, Hat Yai 90110, Songkhla, Thailand; sporncha@medicine.psu.ac.th

**Keywords:** child health, health equity, nutrition status, social determinants, health policy

## Abstract

Background and objectives: Children living in an area distant from or associated with barriers to travelling to health service centres usually experience health and well-being disparities. This is a survey of child health and well-being on Bulon-Don Island, located 22 kms. from the southern mainland of Thailand, to gather essential background data before activating responses from local service provider agencies. Methods: Demographic data, physical and crude psychological health, harm to health, and living conditions of Bulon-Don children aged 1–14 years were studied and compared with the results of the corresponding national child health survey. Descriptive statistics were used for the statistical analysis of significance (*p* < 0.05). Results: A total of 21 male and 41 female children (N = 62) participated in the survey after obtaining consents from parents or care providers. The islanders are Indigenous people who use their own languages and have traditional beliefs. Comparing with the children of the national survey, most children aged <5 years were found to have significantly lower height and weight according to their age (*p* = 0.044 and *p* = 0.043, respectively), whereas those aged >5 years had a similar nutritional status. In addition, there is a lack of facilities for healthy living. However, the mean total psychological and ethical standards scores were significantly higher in the 1–5 and 6–9-year-old children. Conclusions: Disparity of socio-political status, cultural beliefs and practices, socioeconomic basis, and geographic distance from the mainland were the social determinants and barriers of low health service accessibility for the islander children. Comprehensive child health and well-being evaluation in an enclave of isolation like this is mandatory before an integrated intervention carried out by the local healthcare and living facilities providers is implemented.

## 1. Introduction

### 1.1. Background

According to the globally endorsed WHO schemes of ‘Health for all’ and ‘Universal health coverage,’ everyone should access to equitable public healthcare at every societal level, either local, national, or global. In other words, health equity is the avoidance of health inequality between groups of people with different racial, demographic, geographic, socio-economic, and political backgrounds. In addition, health equity can be achieved when everyone attains the full potential for health and well-being [1,2]. However, various socioeconomic, political, and individual demographic as well as health belief factors apparently affect health accessibility, leading to inequity. Although the same health policy has been applied nationwide, intra- or inter-community health inequity has occurred due to distinct demography, socioeconomic factors, and residential geography [3,4,5]. Children, as the youngest members of a society, are highly vulnerable to inequitable healthcare because they absolutely rely on their parents or care providers to seek healthcare. The decision of parents or care providers regarding child healthcare is apparently based on their health literacy to generate rational attitudes and health beliefs in every domain of child care, such as health promotion, disease prevention, acute and chronic disease treatments, and rehabilitation [6]. Given that personal conditions facilitate access to healthcare, some circumstantial factors often deviate the intention and decision to follow health services. Among them, geographic distance from a healthcare centre or living in difficult-to-arrive locations (i.e., an island located in the mid-ocean, dense forest, or high mountain, etc.) is one of several strong determinants of receiving health services, as well as the equal distribution or coverage of public health facilities. Despite a high intention to seek standard healthcare, the unsolved difficulties or barriers in approaching healthcare services prevent healthcare access. When the United Nations’ equitable healthcare concept addresses “leave no one behind,” it means that every individual on earth owns the right to attain standard healthcare wherever he resides. However, geographic distance or difficulties for transportation needing longer travel time to access standard health facilities frequently precludes people from health services, because healthcare-provider sources are usually clustered in urbanised or metropolitan zones. For example, a study from rural Ethiopia revealed that different altitudes in residential areas were inversely associated with the frequency of seeking healthcare, i.e., fewer antenatal care visits and facility deliveries were found in people living at a longer distance or higher altitude from the health facility centre. Therefore, the study suggested that health issues in geographic distance or enclave areas of isolation should be carefully considered in planning universal health coverage policy [7]. Though the right to equitable healthcare access was stressed globally, ease of physical access to a health service facility should be concerned and carefully evaluated as well, because travel distance and inconvenience were potentially key contributors to leaving someone behind in healthcare. Underestimation of influential effects of transportation distance and time factors on accessing healthcare facilities were regarded as a drawback of health policy planning [8,9]. Various distance and travel-time measurement methods were assessed for their performance in estimating travel distance and time required by community members for physical access to a health service facility in Haiti. The study reflected the critical significance of them for equitable healthcare care in the country [9].

Moreover, to achieve goals 4 and 5 of the Millennium Development Goals (MDGs) emphasising the reduction in maternal and childhood mortality [10] and goal 10 of the sustainable development goals (SDGs) targeting health equity [11], the barriers or discouraged factors to access healthcare should be investigated and timely removed. An example of managing the transportation barrier for the full-term pregnant women who resided on an island with no standardised delivery services to undergo facility delivery in a certified child delivery centre on the nearest island among the Philippines archipelago was the use of transporting engine boats owned by the islanders. The applied transporting boat was usually used for communication, fishing, or tourism purposes. Both the boat owner and the child delivery centre where the woman delivered a child could request reimbursement from the national public health fund for maternal and child care [12]. Differences in residential geography apart from the race or political background and socioeconomic status was a factor determining maternal and child healthcare equity. The inequitable attainment of maternal and child healthcare as such eventually resulted in unfavourable child health outcomes [4]. The disadvantaged parents and children living in the rural areas associated with travel difficulties or even the enclaves of isolation in the middle of big cities were highly vulnerable to health access inequity [4,13,14,15,16,17]. Previous studies from low and middle-income countries revealed that geographic-attributed low healthcare accessibility could result in lower facility deliveries leading to poor maternal, neonatal and child health in long term [12,18,19,20,21]. This impactful determinant of healthcare access has been seemingly overlooked in health policy planning, in which local people in these specific areas will be unintentionally left behind. Hence, coherence of policy and operation plan covering health and supporting infrastructures for ease of access to healthcare should be comprehensively considered. The co-designed healthcare plan developed by community dwellers, local healthcare and public facility agencies relating to the well-being of the community dwellers, and particularly the local academic institutions as ‘healthcare partnership’ responsible for ‘academic consultant’ should be initiated and maintained by the local head office of healthcare [22].

It is generally recognised that nurturing a child plays a pivotal role in every social sector and is not limited to the child’s parents and their families. Schools, living communities, local governments, and countries are involved in child health and development outcomes. Hence, integrated child health plans derived from the co-consideration of all stakeholders from the local to the national level are essential. Aiming for the sustainable health goals of a community requires integrative cooperation among all healthcare counterparts to initiate community-targeted programmes [23]. Our study team, as the academic institutional counterpart of child healthcare providers in Satun province, southern Thailand, was informed by the local healthcare volunteers on Bulon-Don Island and the provincial health officers that the islander children had multiple health problems and a lower living standard caused by lacking health knowledge and healthcare resources. However, the directed response of the related provincial governmental agencies has not yet been initiated due to no adequate essential fundamental data on the health and well-being of the children available. Nutritional status and living hygiene were urgent concerns proposed in the initial talks with the island and provincial healthcare officers. Studies showed that prenatal maternal and childhood nutritional status as well as healthy living conditions markedly affected children’s schooling, cognition, physical growth, and psychological development [24,25]. The lack of health knowledge, fewer health professionals and resources distributed to the island due to the geographic distance, and a six-month long yearly monsoon significantly hindered the transportation of well-trained healthcare professionals and resources. These were the contributors to the lower than standard of child healthcare on the island.

### 1.2. Conceptual or Theoretical Framework

The SDGs endorsed by WHO aimed to end poverty and provide healthcare equity regardless of gender, race, political, socioeconomic, cultural, religious, and educational differences. Enjoying a healthy life and environment wherein no one was left behind was the ultimate objective [26]. To achieve good health and well-being (SDG3), various factors contained in the social determinants of health (SDHs)—which include national and regional health policies for equal healthcare access (socio-political context), secure socio-economic position (socio-economic position context), and health beliefs or psychology of community people (intermediary context)—should be carefully considered. When considering the individual items of the SDGs, it is evident that most of them, such as ending poverty (SDG1), zero hunger (SDG2), quality education (SDG4), gender equity (SDG5), clean water and sanitation (SDG6), decent work and economic growth (SDG8), reduced inequalities (SDG10), and sustainable cities and communities (SDG11), align with SDHs. We believe that the SDGs—as health goals, and SDHs—as the determinants to achieve goals could synchronise to yield good health outcomes. In other words, improving SDHs to increase healthcare access and adherence would foster the achievement of the SDGs.

The SDHs are composed of three principal contexts: (a) socio-political context, (b) socio-economic and cultural backgrounds, and (c) intermediary factors as mentioned. Geographic distance or living in a difficult-to-arrive residential location, as in the case of Bulon-Don island in this study, was one of the factors in the socio-political context of SDHs, which included cultural or religious beliefs, clean water supply, sanitation, and life and food security [27,28,29,30]. Ethnicity, gender, education, employment, and socioeconomic status were included in the socioeconomic background, while housing, psychological health, child health, and accessibility to healthcare were included as intermediary factors of SDHs. All three SDH contexts interactively influence people’s attainment of health services [31]. In fact, the determinants of individuals’ health access behaviours were multi-factorial. It was difficult to specify a single most influential one in determining health decisions and behaviours [4]. For these reasons, understanding of the target population’s health and living background in relation to the individual context of SDHs and how they interplay to affect healthcare access is necessary to provide explicit insights before implementing customised health programmes.

Additionally, the development of health programmes to achieve the SDGs introduced by the WHO should cover both direct health and health-promoting facilities to improve both health and well-being. Co-designed programmes among the local stakeholders to secure healthy living should cover economic growth and security, adequate nourishing food, clean piped water and electricity supply, sanitation, and environmental hygiene management beyond education on illness management alone. Academic institutions should support technical knowledge to the local operating agencies in planning and deploying integrated health programmes.

### 1.3. Objectives of the Study

This explorative study aimed to survey both the health and public facilities indicators necessary for the well-being of the islander children. We conducted this multidisciplinary child health survey to evaluate the nutritional background, physical and psychological development, health behaviours, health risk exposure, and public facilities necessary for healthy living available on the island. We aimed to determine the barriers or social determinants that hindered children and their parents from accessing health services, such as socio-political and economic status, traditional cultural beliefs, and transportation. We specifically compared the available study data of Bulon-Don children with those of the National Child Health Survey conducted during the corresponding period to demonstrate the child health disparity between the two populations. As an academic counterpart, we aimed to stimulate the development of relevant tailor-made programmes that incorporate health and well-being issues by provincial healthcare agencies and public facility providers to mitigate the disadvantages of child healthcare on the island.

## 2. Materials and Methods

### 2.1. Study Setting and Design

This study was conducted on Bulon-Don Island, located 22 kms. from the southwestern coast of Satun Province in southern Thailand. It is one of the islands under the coverage of the Petra Islands Marine National Park and the governance of the Paknum subdistrict, La-ngoo district, Satun province. The island has an area of 1659 km^2^, in which about one-third of the total area consists of hills and mountains, while the rest are beaches or seaside areas. The islanders were Urak Lawoi ethnic minorities who believed in Islam, spoke the ancient Malay language, and made their living through traditional fishing along the Andaman Sea coast in southwestern Thailand. Only one local school provided education, with one teacher who taught from primary to fundamental secondary school, and 70 students attended the school. Regarding primary healthcare facilities on the island, there was only one primary healthcare unit (PCU), where two local young adults who graduated from secondary school were selected and trained to provide basic healthcare to the islanders. Severely ill patients or those who required specific medical investigations or treatments were transferred to La-ngoo District Hospital or Satun Provincial Hospital by boat trip, spending at least 2–3 h of transfer time during regular weather conditions. There are two seasons here: summer and rainy seasons, which last longer and have frequent monsoons. During the monsoon days lasting from May to December every year, boat travel to the mainland is impossible. Therefore, the islanders have very limited access to health services and healthcare knowledge.

### 2.2. Study Population, Sample Size, and Sampling Techniques

The study population consisted of children on Bulon-Don Island aged between 1 and 14 years who had been under the care of one of their parents or a care provider for at least 6 months before enrolment. We identified the children and their parents or care providers from the official population registration list on the island. Voluntary participation was the method of enrolment. We included as many voluntary study samples as possible to represent the entire population of children living on the island. Hence, neither sample size nor sampling techniques were applied in this study. After the study details were provided, the children and their parents or care providers were invited to participate in the study based on their voluntary participation. Written informed consent to participate in this study was obtained from the parents or care providers (for children aged <7 years) and from the study children (aged 7–14 years, by ascent forms), together with their parents or care providers, before enrolment into the study process.

### 2.3. Study Methods

After obtaining consent for participation in the study, well-trained data collector staff visited the children’s homes to conduct interviews regarding their health characteristics and health behaviours and observe their housing conditions and surrounding environments. We used well-designed and validated questionnaires to collect the health characteristics and behaviours and nationally validated physical, psychological, and ethical standard assessment tools to collect the data, which were recorded in the designed record form used in the 4th National Child Health Survey by the National Health Commission Office (NHCO).

### 2.4. Study Tools

Physical development was assessed using the standard weighting machine, height metre, and measurement tape validated by NHCO for the national children’s physical development assessment. The psychosocial, emotional, and ethical assessments were performed using the modified assessment tools by the NHCO team for Thais. The tools were also used in the national survey mentioned. They were tested for their content validity by the national experts in the fields of child behaviours and psychological health. Two national experts tested the content validity of Modified Infant Toddler Social and Emotional Assessment (MITSEA) (age 1–5 years) and Modified Emotional Moral Social Assessment (MEMSA) (age 6–9 years) by face validity test, while 5 experts tested Modified Life Skill 1 (MLS1) (age 10–14 years), showing an index of item objective congruence (IOC) of 0.80. The nationwide content reliability test of the three assessment tools reported the Cronbach’s alpha of MITSEA, MEMSA, and MLS1 of 0.8370, 0.8675, and 0.9472, respectively. The time required to complete the individual assessment tool applied in this study was 30 min for MITSEA and 20 min each for MEMSA and MLS1.

### 2.5. Study Variables and Data Analysis

The variables included general demographic data; living conditions of the study children; general health conditions and behaviours; commonly acquired illnesses; vaccination according to the national vaccination programme; physical development indices; and psychosocial, emotional, and ethical standard assessment scores.

The study data of Bulon-Don children were analysed using descriptive statistics, i.e., frequencies, percentages, means (SD), and medians (interquartile range). They were further compared with data from the National Child Health Survey of the NHCO in Thailand to derive statistically significant differences (*p* < 0.05).

## 3. Results

### 3.1. General Demographic Data of the Study Children

Sixty-two children (21 male and 41 female children), who accounted for three-quarters of the total number of children on the island, were enrolled in this study. Children who could not be enrolled were due to following their parents to make a living in other locations or pursuing their studies in schools on the mainland. There were 5, 20, and 42 children aged <2, 1–5, and 6–14 years, respectively. The median number of siblings in each family was three (range 1–7). Their homes were primarily built using single-unit models (93.5%). They had no electricity or piped water for their daily use. A simple electricity generator was used to generate a limited amount of electricity only for nighttime use. Rainwater was the only source of the water supply. They shared public toilets, as there were no private toilets available in individual houses. The average income per family per month was 3500 baht (or 100 USD). The general demographic data were listed in Table 1.

### 3.2. Living Facilities, Sanitation on the Island, Health Behaviours, Illnesses, and Vaccination of Children

Based on interviews and observations during home visits, we summarised the significant health issues and related environmental factors affecting children’s health as follows:

All infants aged <2 years (n = 5) received absolute breastfeeding for the first 3 months, but breastfeeding extended to 6 months was found in only three children. We found that 28.5% of children aged 6–14 years did not complete three meals a day, and breakfast was mostly ignored (11.9%). Among children aged—2–14 years, 39 out of 57 (68.4%) ate vegetables in less than 2 portions per day. However, crispy and unhealthy snacks containing high carbohydrate, salt, and monosodium glutamate (MSG) levels were available on the island.

Two-thirds of the children spent >2 h/day in front of television or personal computer screens, and the duration was much longer among older children, especially on weekends. In addition, 7.1% of 6–14-year-old children played computer games on their personal computers for longer than one hour per day, while children from the other age groups spent less than one hour per day playing on their computers.

We also found that about one-third of the children in the two age groups (26.3% in 6–9 years and 30.4% in 10–14 years) had moderate physical activity (>60 min/day, 5 days/week) in the previous month, and girls exercised at this level of intensity more than boys in both age groups. The median sleep times of children aged 1–5, 6–9, and 10–14 years were 10.7, 10, and 8.9 h, respectively.

Regarding smoking, we found that 4.4% of 6–14-year-old children had experienced smoking, with first-time smoking starting at an average age of 10 years. Interestingly, 73.9% of the children reported passive smoking, and their parents or relatives living in their homes were active smokers. Household smoking was the most common source of passive smoking (70.6%). Owing to Islamic teachings and practices, the islanders absolutely refrained from drinking alcohol.

For illnesses requiring hospitalisation, 10% of 1–5-year-old and 7.1% of 6–14-year-old children were admitted to a hospital on the mainland during the previous month. Respiratory tract diseases, such as asthma, acute bronchitis, and bacterial pneumonia, were common causes of hospitalisation. Only one child aged 1–5 years had been hospitalised because of a traffic accident within the past 12 months.

Seventeen (85.0%) children aged 1–5-year-old received complete vaccination according to the national vaccination schedule. The remaining three children did not complete the vaccination programme. BCG was the most commonly missed vaccine, followed by diphtheria, pertussis, tetanus (DPT), oral polio vaccine (OPV), and Japanese encephalitis (JE) vaccines. This may be attributed to traditional childbirth deliveries at home and no regular visits to a well-baby clinic after childbirth.

### 3.3. Physical Health Measurements

- Height and weight

**Figure 1:**
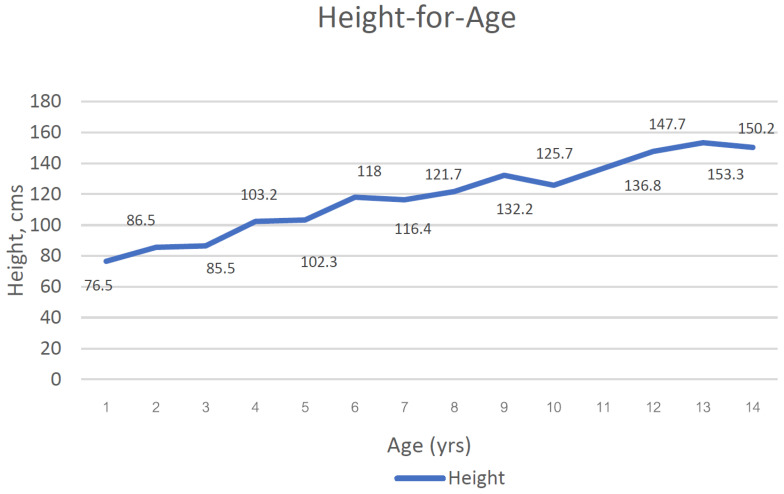
Median height for age of children on Bulon-Don island (yrs., years; cms., centimetres).

**Figure 2:**
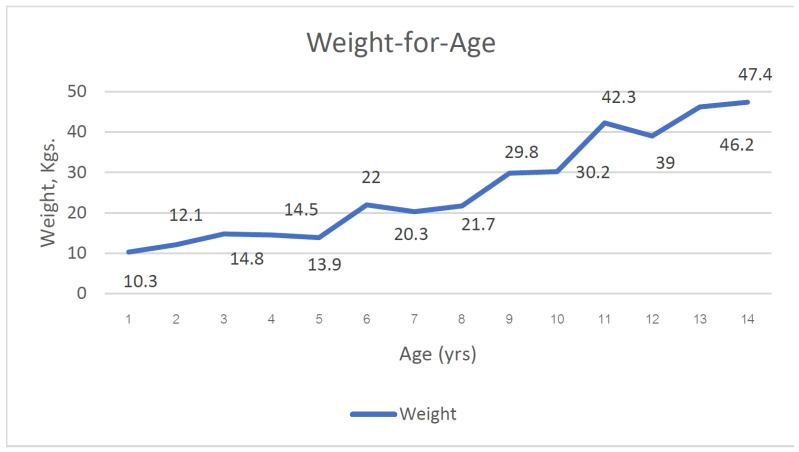
Median weight for age of children on Bulon-Don island (yrs., years; Kgs., kilograms).

The median height and weight for age (HFA and WFA) of Bulon-Don children were shown in Figure 1 and Figure 2.

**Figure 3:**
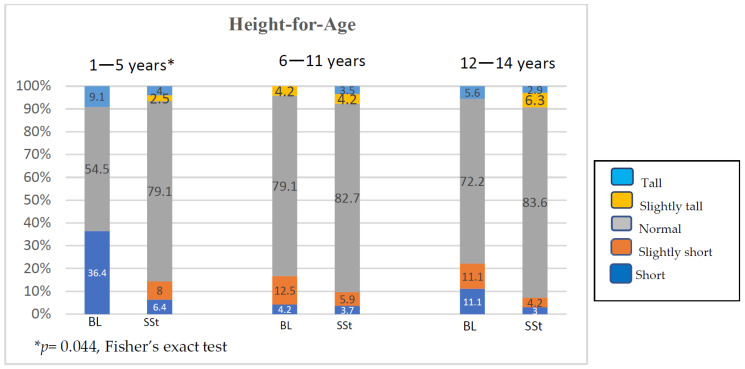
Percentages of children with different height for age in Bulon-Don children compared with those of national survey (BL, Bulon-Don; SSt, national survey).

When the HFA of Bulon-Don children was compared with the results of the national survey, we found significantly higher portion of low HFA in the 1–5 years age group (*p* = 0.044) (Figure 3).

**Figure 4:**
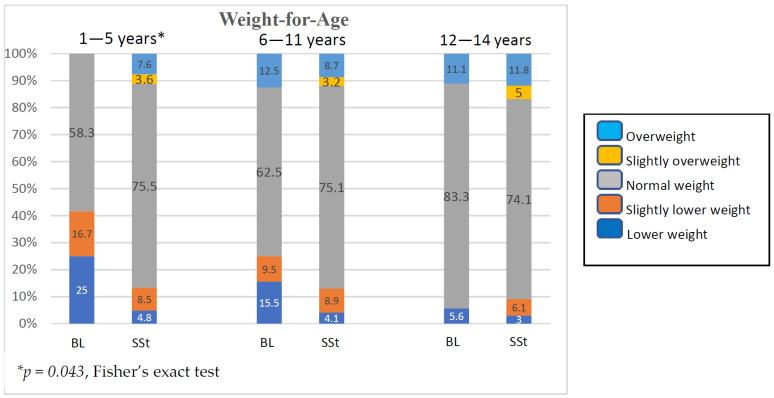
Percentages of children with different weight for age in Bulon-Don children compared with those of national survey (BL, Bulon-Don; SSt, national survey).

Similarly, a significantly higher portion of low WFA was found in children aged 1–5 years (*p* = 0.043). It was also noted that, while Bulon-Don children had a higher percentage of low WFA, children from the national survey had a higher percentage of slightly overweight (Figure 4).

### 3.4. Psychosocial and Ethical Standard Assessments

In this study, we briefly assessed children’s psychological and ethical thoughts. The mentioned assessment tools were applied to assess the psychosocial, emotional, and ethical standards of the children of Bulon-Don island according to age groups. The scores obtained in this study were compared with those of the national survey.

- Age 1 to 5-year-old children

Using the MITSEA tool, we found a significantly higher mean total score for Bulon-Don children than those in the national survey in both sexes (*p* for boys < 0.001, *t*-test; *p* for girls < 0.001, *t*-test).

- Age 6 to 9-year-old children

From the MEMSA tool, we found that the mean total score of Bulon-Don children was significantly higher than that of the national survey in both sexes (*p* for boys = 0.000, *t*-test; *p* for girls = 0.000, *t*-test).

- Age 10 to 14-year-old children

Based on the MLS1 assessment tool, there was no statistically significant difference in total scores between the two groups. Bulon-Don children had higher scores only for some items of the MLS1 tool.

### 3.5. Presentation of Findings and Suggestions to the Local Healthcare and Public Facility Agencies

After the study ended, we presented the findings to the local healthcare and public facility agencies. Suggestions for minimising the disadvantageous determinants for health access causing healthcare inequity occurred on the Bulon-Don children were proposed to local policy makers. The heads and administrative boards of the Paknam District Health Care Office and the Stun Provincial Public Health Office were officially appointed as the leaders of the child healthcare teams. The suggestions for urgent responses to child health and well-being problems on the island were as follows.

(a) Nutritional status of the children starting from breastfeeding to the daily diet of the older children, health risk behaviours, and exposure to unhealthy or harmful substances require healthcare knowledge through effective health education for the parents or childcare providers during receiving regular health services, or group participation actions, as well as for the children while attending classes in school.
(b) Clean water and standard electricity supply, sanitation, and other essential facilities on the island affecting children’s health and well-being should be provided. The immediate distribution of resources and supplies to the island by district and provincial healthcare and public facility agencies is advocated. Furthermore, all health and well-being requirements by the children and childcare providers should be regularly addressed and promptly responded.
(c) Regarding the socioeconomic factors of SDHs, such as household economic and social position, and intermediary factors such as language barriers, religious beliefs or distinct traditional practices, and personal health perception, the islanders require psychosocial educational support and understanding modification without disruption of their traditional beliefs or practices.
(d) For the geographic distance and difficulty in reaching the island, an intermediary context of SDHs, we propose that telehealth education using media contains easily understandable and practicable advice in this situation. The air transfer of healthcare materials and other essential resources possibly overcomes this barrier.

Although highlighting the benefits of attending health services as well as minimising the socio-political determinants (a and b) of access to appropriate healthcare for the children, their parents, or childcare providers is managed, the socioeconomic position and intermediary contexts of SDHs (c and d) of the parents or childcare providers cannot be overlooked. The latter requires supporting policy from local agencies dealing with living facilities to solve these disadvantages.

Since the interplay of the three principal contexts of SDHs affects decisions to access public health services as well as health behaviours, they should be carefully considered when designing a healthcare plan for a specific community. Every sectoral level in the healthcare system, that is, national and local policy makers and community dwellers, should collaboratively participate in health programme design. We believe that this approach can be used in the context of planning health programmes for Bulon-Don children. Furthermore, follow-up child health evaluations and feedback from islanders should be performed and publicised regularly.

We suggested regular team meetings among the related local health and non-health agencies and the research team to follow the progression of programme implementation and outcomes. After our suggestions, we were informed that all the children have received complete vaccination according to the national vaccination programme, and knowledge regarding the benefits of nutrition on the physical and psychological development of children was emphasised. Training to make a living based on the available resources on the island was provided by a local subsection of the Ministry of Social and Human Development. Cooking classes for parents or childcare providers to cook nourishing meals for their children were initiated. Toilets and clean water supply for individual houses are available to most households. The existing PCU on the island was renovated, and healthcare volunteers were retrained and upskilled.

## 4. Discussion

Child health on Bulon-Don island was lower than the national standard because of the lack of knowledge about child nurturing, guidance, and supervision of child healthcare by the local health agencies. At the time of this study, among the mentioned factors, geographic distance leading to transportation difficulty was a major cause of the limited healthcare and living facilities available on the island. Workforce, material, and technical knowledge essential for appropriate health and living were limitedly distributed to the area. Bulon-Don island can be regarded as an enclave of isolation from its neighbourhood by its natural geographic structure. Most of the enclaves of isolation or exclusion by any reason commonly possessed various political, socio-economic, cultural, as well as physical and mental health disparities from their surrounding environments [32]. The attributed socio-economic and low-literate factors resulting from geographic isolation determined the low access to standard child care lead to unhealthy conditions for the islander children. For example, absolute breastfeeding did not last at least 6 months long according to the recommended feeding for infants <2 years of age. Household economic constraints among the islanders could be attributed to shorter absolute breastfeeding time, which significantly affected the physical and psychological development of 1–5-year-old children in this study, compared with the findings of the corresponding national survey (Figure 3 and Figure 4). One study reviewed the threatening nutritional status of 11 countries in Southeast Asia, including Thailand, where wasting and stunting among children < 5 years of age were >5% and >20%, respectively, because of low socioeconomic status and lack of baby-feeding knowledge [33]. Moreover, an undernourished diet during the first 2 years of age was critically associated with childhood stunting and wasting in a study [11]. A study of malnutrition among children under 5 years from a disadvantaged rural district of Pakistan indicated that the longer distance for the community to reach a health facility was associated with higher prevalence of child malnutrition, i.e., OR 2.61 (95%CI 0.85–8.14) for a distance of 3–4 kms, and OR 2.84 (95%CI 0.94–8.82) for a distance exceeding 5 kms [18]. In contrast, a lower morbidity rate related to acute paediatric infections and good child health were significantly associated with closer residency distance from secondary healthcare facilities and maternal and child health centres [20]. The distance to the primary healthcare facility showed a significant reverse correlation with the frequency of visits by the young children living in a rural community of north-western Burkina Faso [19]. Nevertheless, it was found that regular household visits led by women health workers within 15 days to increase healthcare access were associated with the lower prevalence of malnutrition [18]. Based on these findings, outreach for children living in the distant rural communities, ease of transportation to health service centres, or distribution of the essentials for maternal and child healthcare in any way was advocated in the mentioned studies [18,19,20]. To ameliorate child malnutrition in a community, we suggest that the significance of nutrition during prenatal and breastfeeding periods should be strongly emphasised to all women of child-bearing age before conception. An interventional programme to improve parents’ health literacy on this issue is necessary because it can enhance understanding, positive attitudes, and active maternal practices towards breastfeeding. Child healthcare knowledge provided to the community should facilitate the parents or childcare providers to be able to carry out every domain of child care, such as health promotion, disease prevention, treatment of acute or chronic illnesses, and rehabilitation [6]. Meanwhile, socioeconomic agencies should launch parallel strategies to train baby-feeding mothers to make a living sufficiently from their household harvested products or in-house works so that they can breastfeed their babies at home. This approach certainly can sustain the breastfeeding time longer because the household finance is secured. Therefore, up-levelling of health literacy on breastfeeding together with empowering baby-feeding mothers to gain confidence or self-efficacy in longer breastfeeding time by securing household incomes was advocated for promoting child health to achieve SDG3 [34,35].

While a higher percentage of lower WFA was found among children under the age of five in this study (Figure 4), children above 5 years old both on the island and in the national survey were comparably overweight. This could be a model of ‘Double burden of nutrition’ (DBN) in which bimodal malnutrition status, including both under and overnutrition, concurrently occurred in a country [25]. Unhealthy and high-carbohydrate crispy snacks were usually taken to the island by the local grocery-keepers, while high-calorie fast food meals were widely accessible on the mainland throughout the country, causing DBN among the children of the two locations. To date, DBN is considered a global dietary imbalance. While undernutrition is usually found in low socioeconomic communities where a low-nutrient diet causes failure to thrive among infants and early childhood before schooling, overnutrition caused by excessive caloric intake and lack of optimal intensity of physical exercise are common among school-aged children or in an urbanised society [25]. Therefore, knowledge regarding how to balance daily dietary calories should be provided or easily available to all children, their parents, or childcare providers [6]. In addition to high-calorie diets, synthetic chemical food additives have received much attention owing to their adverse effects on child health, particularly among the vulnerable children of low socioeconomic parents [36]. Monosodium glutamate (MSG), a common additive of crispy snacks, was concerned in this study and over the country.

Sanitation and an adequate clean water supply for daily use are also significant predictors of healthy living [37,38]. They are also regarded as factors that connect with various goals in the SDGs [39], particularly SDG3, which stresses a reduction in maternal, neonatal, and child mortality [40,41]. On the island, the volume of water available for daily use relied only on stored rainwater, as no piped water system had been restored. Public toilets were shared, and rubbish disposal systems were not properly managed. Hence, the provincial pipe-water works agency should urgently provide an adequate clean water supply to the island. Advice for full compliance with vaccination according to the national vaccination schedule for children needs to be strengthened for parents or childcare providers. Regarding smoking, the parents or childcare providers should understand the health harm related to the passive smoking condition of their children since it may be underestimated by most adult smokers.

For a brief psychological assessment of the children in this study, we found significantly higher mean scores of psychological and ethical standards among children aged 1–5 and 6–9 years living on the island. Meanwhile, despite no significantly higher mean of overall scores being found, children aged 10–14 years showed significantly higher scores on some items than those in the national survey (MLS1). We presume that the geographically isolated location of Bulon-Don Island prevents the influx of urbanised lifestyles and cultures. However, behavioural or cultural content accessible through personal computers, mobile phones, or television can shape their thoughts when they grow older, as shown by the non-significantly higher mean of overall MLS1 scores (10–14-year-old children). Children should be encouraged to limit their screen time and provided religious and moral teachings by their parents or childcare providers. We observed that most adult islanders retained their traditional lifestyles and strictly followed Islamic teachings. In conclusion, psychological and ethical standards among islander children are favourable based on our brief evaluation. However, the potential changes over time are possible.

Achieving every goal in the SDGs and health for all people regardless of racial, religious, socioeconomic, or political discrimination is the target of health policy makers at all levels. SDHs are powerful facilitators or barriers that determine access to health services. Regarding facilitation of health access for health equity, mindful consideration of the existing SDHs among children and their parents together with amelioration of potential barriers were required [42]. Based on our study, health knowledge that stresses on the significance of every process of child healthcare, starting from regular prenatal visits of pregnant women, facility deliveries, child vaccination, encouraging breastfeeding and good child nutrition, and improving living facilities concurrently with improving transportation between the island and mainland, should be promptly responded to for the health and well-being of islander children. Solutions to the existing or emerging barriers of healthcare access through strengthened collaboration among multidisciplinary local agencies are advocated since attainment of healthy lives is not limited to healthcare policies or interventions alone.

Furthermore, the health belief model (HBM), which is a health psychology construct describing an individual’s intention to adopt or reject a health service available, should be concerned also [43,44,45,46]. Considering the individual context of SDHs consisting of socio-political, socio-economic, and intermediary contexts, we think that HBM can be classified as an intermediary context of SDHs. Therefore, HBM cannot be ignored in promoting standard healthcare access. Achieving universal health coverage in healthcare systems requires multi-level collaboration among global, national, and local agencies, as well as establishing individuals’ rationale beliefs, and willingness to adopt the provided health services, for which HBM plays a vital role here. Healthcare does not limit only to disease diagnosis and treatment but also includes various healthy life-promoting factors such as nutritional security, societal socioeconomic and household financial security, complete immunisation, sanitation, climate change effect control, etc. [47]. Finally, favourable global child health undoubtedly relies on the successful management of individual local child healthcare systems worldwide.

Concerning the healthcare provider side, because of the geographic distance and isolation of Bulon-Don island, in addition to the limited medical resources on the island, distribution and retention of the qualified health professionals to the island were also unavailable. These were the challenges of healthcare security in such geographically compromised areas [4,18,32]. That was why under-qualified local volunteers were trained for performing minor medical and healthcare services for the islanders. A well-structured health service facility was not readily provided to the islander at the time of this study. Telehealth consultations between the healthcare personnel on the island and the main land were expected to relieve the disadvantages. A proper human resources allocation plan should be carefully considered and contained in the master health development policy of the island.

### Strengths and Limitations

The limitations of this study included the small sample size and the cross-sectional study design. However, we enrolled about three-quarters of the children on the island, which could represent all Bulon-Don children for this field survey study. We highlighted the insights into not only child health status but also the SDHs affecting the healthcare accessibility of Bulon-don children. Therefore, while targeting the achievement of SDGs in any communities, evaluating and modifying existing SDHs to encourage equitable access to health services should be concurrently considered.

## 5. Conclusions

Although the health status of islander children is an urgent concern, living facilities for well-being should be co-evaluated. Future studies should thoroughly explore both health and well-being issues of the islander children before drafting a health policy since healthcare is not limited only to health concerns as described before. This explorative study and initial intervention of motivating local multi-disciplinary responses for Bulon-Don children may present the significance of a holistic approach to improve child healthcare on the island. Furthermore, community-specific planning to remove or modify the disadvantaged SDHs for better healthcare access is mandatory through collaboration among all local healthcare counterparts. In summary, coherent strategic planning among the local stakeholders in healthcare combined with the insights on modifying the existing SDHs to facilitate equitable healthcare access is the first and a pivotal step for the achievement.

## Figures and Tables

**Table 1:** General demographic data of the children enrolled in this study.

Variables	n (%), N = 62
Genders	
Male	21 (33.8)
Female	41 (66.2)
Age, years	
1–5 (Male:Female)	6 (30):14 (70)
6–14 (Male:Female)	15(35.7):27(64.3)
Number of siblings/one pair of parents, mean (range)	3 (1–7)
The order of the interviewed child among his/her siblings	
First	21 (33.8)
Second	16 (25.8)
Third	12 (19.4)
Fourth and higher	13 (21.0)
Child care providers	
*Age 1–5 (n = 20)*	
Father and mother	18 (90.0)
Mother alone	1 (5.0)
Close relative	1 (5.0)
*Age 6–14 (n = 42)*	
Father and mother	24 (57.1)
Father alone	3 (7.1)
Mother alone	10 (23.8)
Close relative	5 (12.0)
Marital status of parents	
*Age 1–5 (n = 20)*	
Stay in couple	18 (90.0)
Divorce or separate	1 (5.0)
Dead of either parent	1 (5.0)
*Age 6–14 (n = 42)*	
Stay in couple	32 (76.2)
Divorce or separate	4 (9.5)
Dead of either parent	6 (14.3)
Main occupation of family	
Agriculture	42 (67.7)
Labourers, skilled workers	13 (21.0)
Tourist services	5 (8.0)
Others	2 (3.2)
Parents’ education level	
Father (illiteracy; primary; secondary school)	18 (29); 41(66.2), 3 (4.8)
Mother (illiteracy, primary, secondary school)	14 (22.6); 35 (56.4); 13 (21)

## Data Availability

All study data and analysis methods were included in the article. No whole or parts of the content of this study was deposited in any data depository devices or websites.

## References

[1] World Health Organization (2024). Health Equity in the Western Pacific. https://www.who.int/westernpacific/health-topics/detail/equity.

[2] World Health Organization (2024). Health Equity. https://www.who.int/health-topics/health-equity#tab=tab_1.

[3] Prinja S., Balasubramanian D., Sharma A., Gupta R., Rana S.K., Kumar R. (2019). Geographic Inequities in Coverage of Maternal and Child health Services in Haryana State of India. Matern. Child Health J..

[4] Wahab S., Kelly K., Klingler M., Pirovic A., Futch K., Rennie C., Durham D., Herber D., Gramling G., Price S. (2024). Impact of Race, Socioeconomic Status, and Geography on Healthcare Outcomes for Children With Sickle Cell Disease in the United States: A Scoping Review. Cureus.

[5] Jeon J., Woo A. (2024). Uneven geography of health opportunities among subsidized households:Illustrating healthcare accessibility and walkability for public rental housing in Seoul, Korea. PLoS ONE.

[6] Morrison A.K., Glick A., Yin H.S. (2019). Health Literacy: Implications for Child Health. Pediatr. Rev..

[7] Defar A., Okwaraji Y.B., Tigabu Z., Persson L.Å., Alemu K. (2021). Distance, difference in altitude and socioeconomic determinants of utilisation of maternal and child health services in Ethiopia: A geographic and multilevel modelling analysis. BMJ Open.

[8] Collado Z.C. (2024). The Right to Healthcare Must Include the Right to Ease of Physical Access: Exploring Geography-Health Nexus in GIDA Communities in the Philippines. Int. J. Soc. Determ. Health Health Serv..

[9] Bhangdia K.P., Iyer H.S., Joseph J.P., Dorne R.L., Mukherjee J., Fadelu T. (2022). Comparing absolute and relative distance and time travel measures of geographic access to healthcare facilities in rural Haiti. BMJ Open.

[10] World Health Organization Health in 2015: From MDGs to SDGs. https://www.who.int/data/gho/publications/mdgs-sdgs.

[11] United nations (2023). Global Sustainable Development Reports. https://sdgs.un.org/gsdr/gsdr2023.

[12] The Social Innovation in Health Initiative (2015). Inter-Island Health Service Boat Project. https://socialinnovationinhealth.org/case-studies/inter-island-health-service-boat-project/.

[13] Cyr M.E., Etchin A.G., Guthrie B.J., Benneyan J.C. (2019). Access to specialty healthcare in urban versus rural US populations: A systematic literature review. BMC Health Serv. Res..

[14] Douthit N., Kiv S., Dwolatzky T., Biswas S. (2015). Exposing some important barriers to health care access in the rural USA. Public Health.

[15] Singh G.K., Lee H., Kim L.H., Williams S.D. (2024). Social Determinants of Health Among American Indians and Alaska Natives and Tribal Communities: Comparison with Other Major Racial and Ethnic Groups in the United States, 1990–2022. Int. J. MCH AIDS..

[16] Cortright L., Buckman C., Tumin D., Holder D., Leonard S. (2020). Social Determinants of Health and Emergency Department Use Among Children with Sickle Cell Disease. J. Pediatr. Hematol. Oncol..

[17] Hood A.M., Crosby L.E., Hanson E., Shook L.M., Lebensburger J.D., Madan-Swain A., Miller M.M., Trost Z. (2022). The influence of perceived racial bias and health-related stigma on quality of life among children with sickle cell disease. Ethn. Health.

[18] Shahid M., Ameer W., Malik N.I., Alam M.B., Ahmed F., Qureshi M.G., Zhao H., Yang J., Zia S. (2022). Distance to Healthcare Facility and Lady Health Workers’ Visits Reduce Malnutrition in under Five Children: A Case Study of a Disadvantaged Rural District in Pakistan. Int. J. Environ. Res. Public Health.

[19] Oldenburg C.E., Sié A., Ouattara M., Bountogo M., Boudo V., Kouanda I., Lebas E., Brogdon J.M., Lin Y., for the Étude CHAT Study Group (2021). Distance to primary care facilities and healthcare utilization for preschool children in rural northwestern Burkina Faso: Results from a surveillance cohort. BMC Health Serv. Res..

[20] Corden E., Siddiqui S.H., Sharma Y., Raghib M.F., Adorno W., Zulqarnain F., Ehsan L., Shrivastava A., Ahmed S., Umrani F. (2021). Distance from Healthcare Facilities Is Associated with Increased Morbidity of Acute Infection in Pediatric Patients in Matiari, Pakistan. Int. J. Environ. Res. Public Health.

[21] Fisseha G., Berhane Y., Worku A., Terefe W. (2017). Distance from health facility and mothers’ perception of quality related to skilled delivery service utilization in northern Ethiopia. Int. J. Womens Health.

[22] Steenhoff A.P., Crouse H.L., Lukolyo H., Larson C.P., Howard C., Mazhani L., Pak-Gorstein S., Niescierenko M.L., Musoke P., Marshall R. (2017). Partnerships for Global Child Health. Pediatrics.

[23] Stenberg K., Watts R., Bertram M.Y., Engesveen K., Maliqi B., Say L., Hutubessy R. (2021). Cost-Effectiveness of Interventions to Improve Maternal, Newborn and Child Health Outcomes: A WHO-CHOICE Analysis for Eastern Sub-Saharan Africa and South-East Asia. Int. J. Health Policy Manag..

[24] Assaf S., Pullman T. (2018). Household and Community Risk Factors and Child Well-Being in Low- and Middle-Income Countries. https://ovcsupport.org/resource/household-and-community-risk-factors-and-child-well-being-in-low-and-middle-income-countries/.

[25] World Health Organization (2017). Double Burden of Malnutrition: Policy Brief. https://www.who.int/publications/i/item/WHO-NMH-NHD-17.3.

[26] World Health Organization (2015). Sustainable Development Goals. https://www.who.int/europe/about-us/our-work/sustainable-development-goals.

[27] Pratt C., Taylor R., Smith S.D. (2023). Health Equity and Access to Health Care as a Social Determinant of Health: The Role of the Primary Care Provider. Prim. Care.

[28] Norris K., Jilcott Pitts S., Reis H., Reis H., Haynes-Maslow L. (2023). A Systematic Literature Review of Nutrition Interventions Implemented to Address Food Insecurity as a Social Determinant of Health. Nutrients.

[29] Campanera M., Gasull M., Gracia-Arnaiz M. (2023). Food Security as a Social Determinant of Health: Tackling Inequalities in Primary Health Care in Spain. Health Hum. Rights.

[30] Masotti P., Dennem J., Bañuelos K., Seneca C., Valerio-Leonce G., Inong C.T., King J. (2023). The Culture is Prevention Project: Measuring cultural connectedness and providing evidence that culture is a social determinant of health for Native Americans. BMC Public Health.

[31] World Health Organization (2010). A Conceptual Framework for the Action on Social Determinants of Health 2010. https://iris.who.int/bitstream/handle/10665/44489/9789241500852_eng.pdf?sequence=1.

[32] Źróbek-Różańska A. (2020). Enclaves of Isolation and Neglect in Rural Areas. Evidence from North-Eastern Poland. Land.

[33] Nguyen T.T., Darnell A., Weissman A., Cashin J., Withers M., Mathisen R., Lapping K., Mastro T.D., Frongillo E.A.A. (2020). National nutrition strategies that focus on maternal, infant, and young child nutrition in Southeast Asia do not consistently align with regional and international recommendations. Matern. Child Nutr..

[34] Dewey K.G. (2016). Reducing stunting by improving maternal, infant and young child nutrition in regions such as South Asia: Evidence, challenges and opportunities. Matern. Child Nutr..

[35] Onah M.N. (2020). Women’s empowerment and child nutrition in South-Central Asia; how important is socioeconomic status?. SSM Popul. Health.

[36] Trasande L., Shaffer R.M., Sathyanarayana S., Council on Environmental Health (2018). Food Additives and Child Health. Pediatrics.

[37] Torlesse H., Aguayo V.M. (2018). Aiming higher for maternal and child nutrition in South Asia. Matern. Child Nutr..

[38] Yarparvar A., Jewell J.M., Al-Jawaldeh A. (2019). Regional Overview on Maternal and Child Nutrition and Examples of Nutrition Governance and Policy Responses: Europe, Central Asia and Eastern Mediterranean Regions. Ann. Nutr. Metab..

[39] Omidakhsh N., von Ehrenstein O.S. (2021). Improved Water, Sanitation and Utilization of Maternal and Child Health Services in South Asia-An Analysis of Demographic Health Surveys. Int. J. Environ. Res. Public Health.

[40] UNESCO (2020). World Water Assessment Programme. The United Nations World Water Development Report 2020: Water and Climate Change.

[41] United Nations (2015). Transforming Out World: The 2030 Agenda for Sustainable Development.

[42] Pearce A., Dundas R., Whitehead M., Taylor-Robinson D. (2019). Pathways to inequalities in child health. Arch. Dis. Child..

[43] Damghanian M., Mahmoodzadeh H., Khakbazan Z., Khorsand B., Motaharinezhad M. (2020). Self-care behaviours in high-risk women for breast cancer: A randomized clinical trial using health belief model education. J. Educ. Health Promot..

[44] Sadeghi R., Tol A., Moradi A., Baikpour M., Hossaini M. (2015). The impacts of a health belief model-based educational program on adopting self-care behaviours in pemphigus vulgaris patients. J. Educ. Health Promot..

[45] Thahirabanuibrahim Logaraj M. (2023). The effect of the health belief model education for cervical cancer prevention, screening promotion among rural women in Chengalpattu district, Tamil Nadu (HBMECC). J. Educ. Health Promot..

[46] He L., Gao S., Tao S., Li W., Du J., Ji Y., Wang Y. (2020). Factors associated with colonoscopy compliance based on Health Belief Model in a community-based colorectal cancer screening program Shanghai, China. Int. Q. Community Health Educ..

[47] Buser J.M., Taha A.A. (2022). Global Child Health Is Local Child Health. J. Pediatr. Health Care.

